# Counteracting learned non-use in chronic stroke patients with reinforcement-induced movement therapy

**DOI:** 10.1186/s12984-016-0178-x

**Published:** 2016-08-09

**Authors:** Belén Rubio Ballester, Martina Maier, Rosa María San Segundo Mozo, Victoria Castañeda, Armin Duff, Paul F. M. J. Verschure

**Affiliations:** 1 Laboratory of Synthetic, Perceptive, Emotive and Cognitive Systems, Universitat Pompeu Fabra, Roc Boronat 138, Barcelona, Spain; 2 Servei de Medicina Física i Rehabilitació del, Hospital Universitari Joan XXIII de Tarragona, Tarragona, Spain; 3 Institució Catalana de Recerca I Estudis Avançats (ICREA), Barcelona, Spain

**Keywords:** Stroke, Rehabilitation, Deductive medicine, Learned non-use, Virtual reality

## Abstract

**Background:**

After stroke, patients who suffer from hemiparesis tend to suppress the use of the affected extremity, a condition called learned non-use. Consequently, the lack of training may lead to the progressive deterioration of motor function. Although Constraint-Induced Movement Therapies (CIMT) have shown to be effective in treating this condition, the method presents several limitations, and the high intensity of its protocols severely compromises its adherence. We propose a novel rehabilitation approach called Reinforcement-Induced Movement Therapy (RIMT), which proposes to restore motor function through maximizing arm use. This is achieved by exposing the patient to amplified goal-oriented movements in VR that match the intended actions of the patient. We hypothesize that through this method we can increase the patients self-efficacy, reverse learned non-use, and induce long-term motor improvements.

**Methods:**

We conducted a randomized, double-blind, longitudinal clinical study with 18 chronic stroke patients. Patients performed 30 minutes of daily VR-based training during six weeks. During training, the experimental group experienced goal-oriented movement amplification in VR. The control group followed the same training protocol but without movement amplification. Evaluators blinded to group designation performed clinical measurements at the beginning, at the end of the training and at 12-weeks follow-up. We used the Fugl-Meyer Assessment for the upper extremities (UE-FM) (Sanford et al., Phys Ther 73:447–454, 1993) as a primary outcome measurement of motor recovery. Secondary outcome measurements included the Chedoke Arm and Hand Activity Inventory (CAHAI-7) (Barreca et al., Arch Phys Med Rehabil 6:1616–1622, 2005) for measuring functional motor gains in the performance of Activities of Daily Living (ADLs), the Barthel Index (BI) for the evaluation of the patient’s perceived independence (Collin et al., Int Disabil Stud 10:61–63, 1988), and the Hamilton scale (Knesevich et al., Br J Psychiatr J Mental Sci 131:49–52, 1977) for the identification of improvements in mood disorders that could be induced by the reinforcement-based intervention. In order to study and predict the effects of this intervention we implemented a computational model of recovery after stroke.

**Results:**

While both groups showed significant motor gains at 6-weeks post-treatment, only the experimental group continued to exhibit further gains in UE-FM at 12-weeks follow-up (*p*<.05). This improvement was accompanied by a significant increase in arm-use during training in the experimental group.

**Conclusions:**

Implicitly reinforcing arm-use by augmenting visuomotor feedback as proposed by RIMT seems beneficial for inducing significant improvement in chronic stroke patients. By challenging the patients’ self-limiting believe system and perceived low self-efficacy this approach might counteract learned non-use.

**Trial registration:**

Clinical Trials NCT02657070.

**Electronic supplementary material:**

The online version of this article (doi:10.1186/s12984-016-0178-x) contains supplementary material, which is available to authorized users.

## Background

After stroke, a neural shock leads to a learning process in which the brain progressively suppresses the use of the affected extremity [1]. This phenomenon is commonly referred to as learned non-use [2, 3]. Constraint-Induced Movement Therapy (CIMT) [1] implements a technique that aims to re-integrate the affected arm in the performance of Activities of Daily Living (ADLs) and reduce learned non-use. In order to achieve this goal, CIMT proposes to restrict the movement of the patient’s less-affected arm for about 90 % of the patient’s waking hours, which physically forces the use of the affected arm during performance of ADLs. Although a number of studies have shown the effectivity of CIMT [4], the high intensity of its protocols severely compromises its adherence [5] and can be physically and mentally tiring [6]. Moreover, its application is restricted to patients without severe cognitive impairments and with mild hemiparesis, which only accounts for about 15 % of all stroke cases [7]. Due to this limitations, several studies have tested variants of CIMT with reduced intensity protocols, giving rise to a Modified Constraint-Induced Movement Therapy (mCIMT) [8] and the so called Distributed Constraint-Induced Movement Therapy (dCIMT) [9]. However, the inclusion criteria of this type of therapy still remains excessively stringent [8, 10], and its efficacy at the chronic stage is unclear [11]. Given these limitations, there is a need for developing alternative methods that build on CIMT principles to foster the usage of the paretic limb, while mitigate its limitations.

A better understanding of the different factors determining hand selection could provide valuable insights for the development of new treatments that effectively counteract learned non-use and promote functional recovery. Previous studies have shown that the history of rewards may strongly bias action selection and habit learning [12–15]. Indeed, perceived self-efficacy, i.e. one’s own belief in his or her capabilities to successfully execute actions that are required for a desired outcome [16], appears to be an important driver for health behavior improvements [17]. In addition, the minimization of the expected cost/effort associated to a given action may as well regulate the decision making process [18]. The strong influence of these two factors on hand selection (i.e. expected cost and expected reward) may be sufficient to approximate the prediction of hand selection patterns, and may provide a direct explanation of our general preference for the execution of ipsilateral movements [19]. Following this line of research, we have shown in previous studies that hemiparetic stroke patients may be highly sensitive to failure when using the affected limb, therefore exposure to goal-oriented movement amplification in VR when using the affected extremity may serve as implicit reinforcement and promote arm use [20]. The resulting bias in hand selection patterns may rapidly emerge via action selection mechanisms, both reducing the expected cost and increasing the expected outcome associated to those movements executed with the paretic limb. It is generally known that motor learning is driven by motor error, and the high redundancy of the human motor system allows for the optimization of performance through decision making processes (i.e. effector selection). Thus, by virtually reducing sensorimotor error, these decision making processes can be modulated through intrinsic evaluation mechanisms [21, 22]. Previous studies have further proposed that a successful action outcome might reinforce not only the intended action but also any movement that drives the ideomotor system during the course of its execution [23–25]. This theory suggests that accidental success after action selection may be an effective mechanism for the spontaneous emergence of compensatory movements [26]. On this basis, by reducing sensorimotor feedback of those goal-oriented movements performed with the paretic limb, we may reinforce the future selection of the executed action. Indeed, a fMRI study on one stroke patient suggests that activations in the sensorimotor cortex of the affected hemisphere (the “inactive” cortex) were significantly increased simply by providing feedback of the contralateral hand [27]. This effect was also observed in healthy subjects [27]. In more recent studies, the effect of visuomotor modulations in motor adaptation has been also explored, showing that diminished error feedback and goal-oriented movement amplification does not necessarily compromise error-based learning [22, 28]. Building on these findings and grounding them on the Distributed Adaptive Control (DAC) theory of mind and brain, which proposes that restoring impaired sensorimotor contingencies is the key for promoting recovery [29], we propose a new motor rehabilitation technique that we term Reinforcement-Induced Movement Therapy (RIMT) [20]. This strategy is a combination of the following methods: 1) Shaping through training, while increasing the task difficulty according the patient’s performance; 2) limiting the use of the non-affected arm by introducing contextual restrictions in VR (i.e. restricted and symmetrically matched workspace for each arm); 3) providing explicit feedback about performance to the patient; and 4) augmenting goal-directed movements of the paretic limb in virtual reality (VR), in such a way that the patient executing the movement is exposed to diminished visuomotor errors, both in terms of distance and directional accuracy, thus increasing the expected action outcome (i.e. expected success) and decreasing the expected action cost (i.e. expected effort) [21]. While principles one to three of RIMT are similarly present in CIMT and Occupational Therapy protocols, the novelty of RIMT resides in its fourth principle: the provision of implicit reinforcement through the reduction of sensorimotor errors. This unique component of RIMT is the only variable that will be manipulated in the present study.

We hypothesize that by reducing visuomotor error within RIMT protocols, we may be able to boost the patients’ perceived performance of the paretic limb, leading to an increased use over time. Consequently, the increased spontaneous use of the paretic limb may facilitate intense practice and induce use-dependent plastic changes, therefore establishing a closed loop of recovery in which arm use and motor recovery reinforce each other. In this vein, a recent computational model of motor recovery suggested that there may be a functional threshold that predicts the use of the paretic limb after therapy [13, 30]. According to this model, only therapies that enable the patient to exceed a given functional threshold will recursively increase the spontaneous use of the paretic limb and induce functional improvement, leading to a complete motor recovery. This principle of *use it or loose it* can as well predict the effectiveness of RIMT. Furthermore, based on simulations from a computational model, we propose that reinforcement-based and constraint-based protocols can be combined to maximally promote the use of the paretic limb and induce functional gains in the chronic phase after the stroke. To test our hypothesis we conduct a randomized, double-blind, longitudinal clinical study with chronic stroke patients, and we analyze the effects of RIMT intervention on counteracting learned non-use and inducing motor recovery.

## Methods

In the following section we first briefly describe a computational functional model of motor recovery after stroke that grounds our hypothesis, next we present a behavioral clinical study with chronic stroke patients.

### Theoretical grounding

In order to study the effects and possible applications of reinforcement-based therapies, we implemented a computational model of recovery after stroke to simulate different variations of RIMT and CIMT combinations. The model thus allowed us to study optimal combinations of these two therapies for an effective rehabilitation. Recently the influence of arm use on motor recovery has been explored through a bi-stable model of motor recovery after stroke developed by [13]. This functional model simulated planar unimanual reaching movements towards a target. In this simulations, movement outcome informed the system to maximize future performance. We extended this model by integrating the planing of movement extent as an indicator of motor performance, and by incorporating the expected cost of a movement as a parameter for action selection. Detailed description of the model can be found in Additional file 1, section *Computational model description* and has been published elsewhere [20]. Simulations showed that the averaged probability of choosing the paretic limb and mean directional errors across trials increased in both constraint-based and reinforcement-based treatment conditions (Additional file 1, section *Results from the model*). We identified a threshold of performance and a threshold of arm use that initiated a virtuous loop of recovery by promoting the spontaneous use of the paretic limb. This bistable dynamics induced further performance improvement and restored typical hand selection patterns at follow-up. Contrarily, the no-therapy condition progressively discouraged the use of the affected limb and predicted further deterioration.

### Experimental protocol and set-up

#### Subjects

From January 2014 until May 2015, 23 hemiparetic stroke patients from Hospital Universitari Joan XXIII in Tarragona, Spain, were recruited according to the following inclusion criteria: a) patients with upper-limb hemiparesis due to a first-ever ischemic or hemorrhagic stroke (at least > four weeks post-stroke); b) between 25 and 75 years old; c) demonstrating an upper limb motor deficit superior to two points as measured by the Medical Research Council Scale for proximal muscle strength; d) a spasticity in the affected upper limb of less than three points as measured through the Modified Ashworth Scale; e) sufficient cognitive capacity to be able to follow the instruction of the intervention training as measured through the Mini Mental State Evaluation (superior than 24 on the scale). Exclusion criteria were defined as: a) severe cognitive deficits that impede the correct execution or understanding of the intervention training; b) severe impairments in vision or visual perception abilities (such as vision loss or spatial neglect), in spasticity, in communication abilities (such as aphasia or apraxia), severe pain as well as other neuromuscular or orthopedic changes that impede the correct execution of the intervention training; d) mental dysfunctions during the acute or subacute phase after the stroke. All patients were right-handed.

The study was approved by the local Ethical Committee at Hospital Universitari Joan XXIII, and the written consent to participate in the experiment was obtained from all patients involved.

The 23 patients were recruited through the administrative staff of the rehabilitation center of the Hospital Universitari Joan XXIII and then randomly assigned to two groups, an Experimental Group (EC) or a Control Group (CG), by the experimenter who ensured a balanced allocation in the two groups (see Additional file 2). Patients’ demographics and characteristics are shown in Table 1. Clinicians, that were blinded regarding the group allocation, conducted the clinical assessments at the beginning of the experiment (baseline, T0), after six weeks at the end of the treatment (T1) and at follow-up after 12 weeks (T2). The experiment concluded in August 2015. Patients were instructed not to follow any specific therapy during the participation period.

**Table 1:** Patient’s characteristics at baseline (*n*=18)

Characteristics	EG n (%)	CG	*p*-values
Subjects	12 (52 %)	11 (48 %)	
Dropouts	3 (13 %)	2 (9 %)	
Compliants	9 (39 %)	9 (39 %)	
Gender			.578
Female	2 (11 %)	1 (6 %)	
Male	7 (39 %)	8 (44 %)	
Etiology			1.000
Hemorrhagic	1 (6 %)	3 (17 %)	
Ischemic	8 (44 %)	6 (33 %)	
Lesion side			1.000
Right	5 (28 %)	4 (22 %)	
Left	4 (22 %)	5 (28 %)	
Mean (SD) - Median			
[25th–75th percentiles]			
Age, years	63.40 (9.40) – 63	54.80 (12.00) – 57	.154
	[57.80–68.50]	[50.80–63.30]	
Days	1298.44 (1968.48) – 400	1387.33 (1455.12) – 735	.232
poststroke	[269.25–1373.00]	[493.50 – 1826.00]	
Clinical scales			
Total UE-FM	32.33 (16.09) – 38	36.89 (12.29) – 40	.651
	[25.50–40.75]	[50.80–63.30]	
UE-FM-Proximal	17.00 (7.40) – 17	18.89 (6.01) – 19	.88
	[12.50–21.50]	[16.88–21.13]	
UE-FM-Wrist	5.78 (3.60) – 8	4.78 (3.31) – 5	.49
	[5.75–10.25]	[2.25–7.75]	
UE-FM-Hand	7.44 (4.69) – 8	11.44 (4.72) – 12	.15
	[4.63–11.38]	[8.50–15.50]	
UE-FM-	2.56 (1.67) – 3	2.78 (1.30) – 3	.99
Coordination	[1.75–4.25]	[2.00–4.00]	
CAHAI	32.56 (14.47) – 36	36.89 (12.29) – 40	.475
	[25.50–42.25]	[16.00–45.00]	
BI	85.33 (10.82) – 88	90.56 (7.32) – 90	.445
	[80.00–91.00]	[84.00–96.25]	
Hamilton	14.44 (9.61) – 8	12.44 (9.10) – 10	.649
	[6.75–24.75]	[5.50–19.50]	

Statistical test used for *p*-value: Wilcoxon rank-sum test

From the 23 patients recruited, five were excluded due to the following reasons: a) two patients presented spatial neglect; b) two patients that were assigned to EG, failed to complete the intervention training of six weeks; and c) one patient dropped out after the recruitment. The final analysis was therefore performed on a total of 18 patients (*n*=18), nine in each group.

#### The Rehabilitation Gaming System (RGS)

In order to provide RIMT as an intervention for motor recovery, we used the Rehabilitation Gaming System, a virtual reality-based rehabilitation tool that has shown to be a valid approach to provide augmented multimodal feedback and effective sensorimotor training in clinical setups [21, 31, 32]. RGS incorporates the neurorehabilitation paradigm that action execution and observation of the same action might activate the functional reorganization of the motor and pre-motor systems that are affected by a stroke, potentially by recruiting undamaged primary or secondary motor areas through alternative sensorimotor pathways [33]. This can be achieved as the patient controls with his own movements a virtual body (avatar) on a computer screen and observes the digital movement from the first-perspective. By modulating this visuomotor feedback we can provide goal-oriented movement amplification in VR, consequently exposing the subject to diminished errors.

#### Set-up

**Fig. 1:**
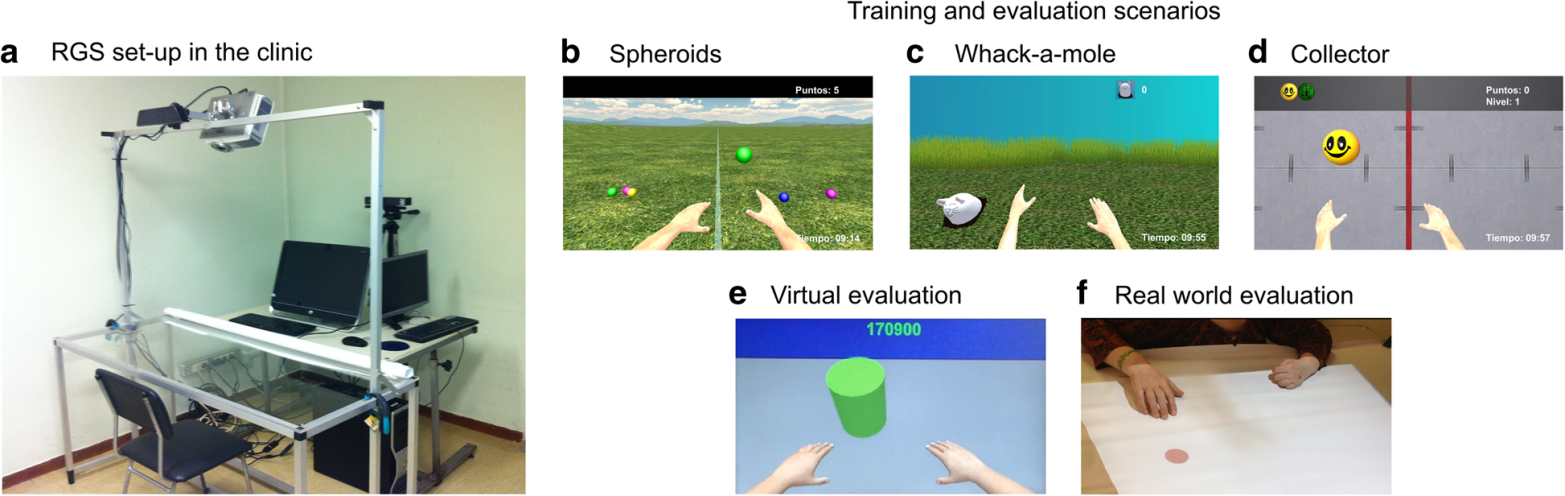
Set-up and scenarios. **a** RGS setup in the hospital showing the transparent acrylic table in front of which the desktop computer with the Kinect (on a tadpole that elevates it above the screen) is placed. In order to use the second Kinect and the overhead projector on the scaffold above the table for the real world evaluation scenario, a white cover can be placed over the acrylic surface. During a training session, the user sits in a chair facing the screen while resting his/her arms on the table. **b** Spheroids scenario, where sets of colored spheres are launched towards the player who has to intercept them. **c** Whack-a-mole scenario, where the user freely chooses which limb to use in order to reach towards an appearing mole. **d** Collector scenario, where a set of patterned spheroids as indicated in the upper-left corner of the screen need to be collected. **e** Virtual evaluation scenario, an abstract version of the Whack-a-mole scenario, where the patient has to reach towards an appearing cylinder. **f** Real-world scenario, where the user has to reach towards randomly appearing dots that are projected from above on the table surface in front of him or her

The clinical set-up of RGS consisted of a desktop touch screen computer with integrated CPU that displays the scenarios to the patients and a Microsoft Kinect motion capture system (Microsoft, US) for tracking the upper-limb movement of the patient and mapping it to the virtual arms of an avatar. The computer and the Kinect were placed in front of an acrylic table that allowed the patients to rest their arms during the session (Fig. 1a). In addition, a metallic frame was placed on top of the table, where a second Kinect and an overhead projector facing the table were mounted. This additional set-up was needed for one of the evaluation scenarios that are described after the following section.

#### Training scenarios

The three training scenarios used in this study (Fig. 1b-d) which are called Spheroids, Whack-a-mole and Collector were game-like intervention protocols that incorporated various features that aimed to promote the usage of the paretic limb, either forced or voluntarily. In the Spheroids and Collector scenarios the patients were required to intercept colored or patterned spheres by performing horizontal lateral arm movement. A bar in the middle of the scenery split the virtual workspace in two sides, herewith forcing the patient to perform ipsilateral movements only; targets that appeared in the paretic side of the screen had to be intercepted with the paretic limb, whereas the less-affected limb could only be used for the targets that appeared in the workspace ipsilateral to the less-affected side. As targets could occasionally appear simultaneously in both work spaces, the patient was prompted to do bi-manual training. Since the avatar’s arm movement was controlled by the patient’s joints of the upper extremities and the avatar’s arm length was fixed, the distance from the avatar’s hand to the target was equal across patients. For every successfully intercepted sphere the patient was rewarded with a point. Within the Collector scenario the spheres fell from the upper part of the screen to the bottom, where the patients could intercept them. In contrast to Spheroids [34], did the Collector scenario possess an additional cognitive component. In the third scenario themed Whack-a-mole, patients executed a horizontal reaching movement to eliminate targets (moles) that appeared sequentially on a planar surface. The location of the target did not determine which hand had to be used, the patients were free in choosing one or the other limb for any given target, therefore applying ispi- and contralateral movements. In contrast to the other scenarios the hands had to be placed on start positions, that were indicated by two red cylinders of 7.5 cm in diameter and that were located 48 cm apart from each other, to initiate the appearance of a target respectively a trial. The hands had to be maintained on the start positions for a variable time of 1 ±0.5 seconds, after which the start positions disappeared and a target was generated. The target could be located at any of nine possible positions that were defined in angles from the body mid-line (0, ±4, ±8, ±6 and ±32 degrees), forming a hemicircle on the planar surface, that was 65 cm away from the avatars body. In this scenario the maximal visibility of the target was set to 1.75 seconds, therefore setting a time limit for reaching, while the pace in Spheroids and Collector was only given by the speed of the approaching spheres. If the patient successfully reached for the target within this time limit, the target disappeared and the patients was rewarded with a score that incremented by 30 points for each tenth of a second as the virtual hand was held over the target’s position.

In all training scenarios the movements to be performed were planar and were executed over a surface providing anti-gravity support. The task difficulty was adjusted automatically to the performance level of the patients in order to provide a customized and balanced rehabilitation experience that posed an optimal challenge level to the patients. A detailed explanation of the automated difficulty mechanisms can be found elsewhere [35]. The parameters adjusted automatically within the Spheroids scenario were the speed, the size and the range of the appearing sphere. Within the Collector scenario only the speed parameter and within the Whack-a-mole only the size parameter of the targets were adjusted automatically. Common in all scenarios was that success and failure were indicated with a respective sound as well that points were displayed during the game in the upper right corner of the screen and were reset after each daily session. Besides that all scenarios provided motor training, Spheroids and Collector forced the patients to use their paretic limb for targets in the given workspace, whereas Whack-a-mole served as a tool to evaluate hand selection patterns.

#### Evaluation scenarios

Before the start of the training sessions and at the end of every week, the groups completed two additional evaluation scenarios (Fig. 1e–f). The first virtual evaluation scenario was an abstract version of the Whack-a-mole scenario, but where no movement amplification was applied in any group, and the trials were fixed to a given amount of targets per angle in the semicircle array. The second evaluation scenario tested the hand selection pattern of both groups in a real world scenario. In this scenario, that was inspired by the Bilateral Arm Reaching Test (BART) of a study by Han et al. [36], the patients had to reach physically for randomly appearing dots that were projected from the overhead projector on the table. The movement of their limbs was tracked with the Kinect that was mounted next to the overhead projector. The targets were arranged similarly as in the Whack-a-mole scenario in four semicircle arrays with angles of ±5, ±15, ±25, ±35, ±45, ±55, ±65, ±75 and with radii of 21, 27, 33 and 39 spreading out from the body mid-line of the patients. The patients were free in selecting one limb or the other for a given target. As in the Whack-a-mole game there were two start positions where the hands had to be placed in order to start a trial. This evaluation scenario was used to assess whether acquired hand selection patterns translated from the virtual space into a real world set-up. No feedback on success or failure was given to the patient.

#### Intervention

**Fig. 2:**
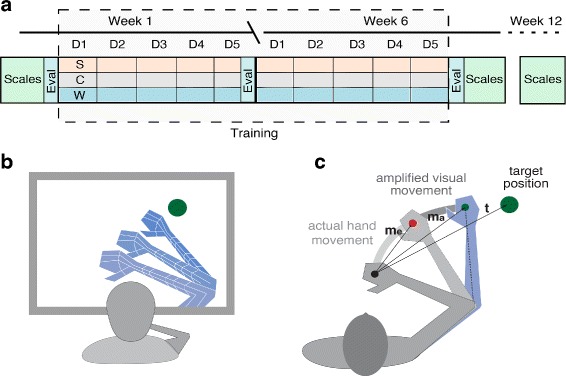
Experimental protocol. **a** Experimental condition: during training the participant visualizes augmented goal-oriented movements that match his/her intended actions. **b** Diagram showing the methodology for the amplification of goal-oriented reaching movements in VR. At each time step, the executed movement vector is attracted towards the target, both in terms of extent and direction. **c** The clinical assessments (*light green*) are performed before the training, at the end of the training and at 12 weeks follow-up. The virtual and the real world evaluation (*dark green*) are performed at the beginning of the treatment and at the end of every training week. Every workday for six weeks all patients completed a session containing the three training scenarios in the following order: Spheroids (S), Whack-a-mole (W) and Collector (C)

Both groups EG and CG were asked to perform 30 training sessions over the course of six weeks (one session a day, for five days a week, Fig. 2a). One session consisted of playing every scenario once for 10 min (30 min in total per training session). However in the EG group we modified the visuomotor feedback that the patients received while training. Undisclosed to the EG subjects, we applied a movement amplification on the virtual representation of the paretic limb that led to a reduced exposure to visuomotor error feedback (Fig. 2b), whereas no such modulation was applied in the CG. The movement amplification took the patient’s movement with the paretic limb and instead of mapping it one to one on the virtual limb of the avatar, augmented it both in accuracy and extent before it was applied to the digital representation (Fig. 2c). At each frame, while the patient progressed in the scenario, we obtained from the Kinect a vector (*m*) of the currently executed movement with the paretic limb and multiplied it by a constant gain factor *G*. The resulting vector (*m*_*e*_) was projected towards the vector of the target (*t*), from which we obtained the direction vector (*m*_*p*_). Finally the exact amount of augmentation in the current time frame was calculated:

1 $$\begin{array}{@{}rcl@{}} & m_{a} &= \alpha\cdot m_{p}+(1-\alpha)m_{e}\\ where&\alpha&=\frac{|m_{p}|}{|t|}\cdot H \end{array} $$

where *H* was a constant help factor. Notice that the movement amplification vector *m*_*a*_ was a weighted combination of two terms: an accuracy amplification vector and an extent amplification vector. The amount of contribution of each of these two components was determined by the *alpha* ratio. After computing the movement amplification vector *m*_*a*_, the theoretical hand position in the virtual space could be extracted. By applying an inverse kinematics technique (Cyclic Coordinate Descent) the corresponding elbow and shoulder joint could be determined. As a last step these estimates were mapped on the virtual representation of the paretic limb. The constant factor *G* was set to 1.4 and *H* was fixed to 0.7.

### Outcome measures

Outcome measurements were taken from four standard clinical scales, that were assessed before (T0) and at the end of the treatment (T1) as well as at 12-weeks follow-up (i.e. 6 weeks after the end of the treatment) (T2). Additional measurements regarding arm use were extracted from the scenarios. The primary outcome measurement consisted of the upper extremity Fugl-Meyer Assessment (UE-FM) [37] and its subscales for Proximal, Wrist, Hand and Coordination function. Secondary outcome measurements were the outcomes of the remaining clinical scales: Chedoke Arm and Hand Activity Inventory to evaluate changes in bi-manual motor function (CAHAI-7) [38], Barthel Index to assess effects in functional independence (BI) [39], Hamilton to assess changes in mood disorders [40], and the calculation of the change in hand selection patterns in the training and evaluation scenarios.

### Statistical analysis

The homogeneity of the two groups at baseline with regards to demographic measures, stroke characteristics and clinical scales was assessed using the Wilcoxon rank-sum test (Table 1). Homogeneity between groups at baseline was confirmed for all measurements (Table 1).

**Fig. 3:**
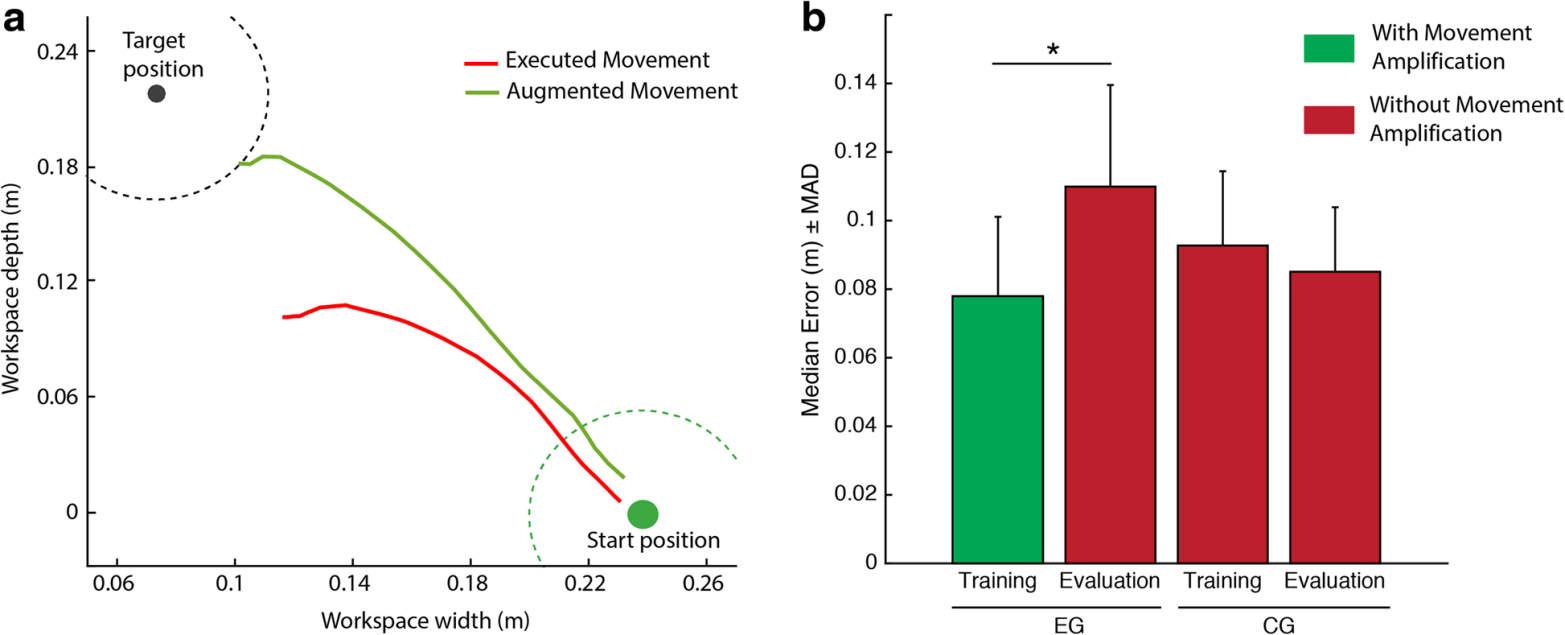
Validation of the movement amplification mechanism. **a** Example trajectory of the patient’s real arm movement (*red curve*) and the amplified movement in VR (*green curve*). **b** Median of reaching errors (i.e. distance from the center of the avatar’s hand to the target) of the virtual movement by group and scenario. Error bars indicate median absolute deviations for each group

In order to verify that the movement amplification mechanism indeed reduced visuomotor error, we first quantified the mean error per session and subject, both in the training and evaluation scenarios. Error was defined as the minimum distance from the avatar’s hand to the target location along each trial. Next we performed a within-subject analysis comparing mean errors in the training scenario (i.e. with movement amplification) and the evaluation scenario (i.e. without movement amplification) by applying a Wilcoxon rank-sum test. Our analysis revealed that the method we used for the amplification of goal-oriented movements reduced significantly the magnitude of the error experienced by the EG during training (median −0.07, MAD 0.037, *p*<.01, *r*=−0.62, Fig. 3).

In order to analyze the clinical impact of the intervention (independent variable: augmented goal-directed movement or absence of augmentation) on the clinical measurements (dependent variable: primary and secondary outcome measurements) over time, we calculated for each patient the change from the baseline measurements (T0) to the measurements at the end of training (change at T1: T1-T0) and to the measurements at 12-weeks follow-up (change at T2: T2-T0). The descriptive data for each scale can be found in Table 2. In order to test for significant within-group effects at each time step, a Wilcoxon signed-rank test was used. In order to compare the changes at T1 and at T2 between groups, a Wilcoxon rank-sum test was applied. As normality tests (Lilliefors test) revealed that only the changes in UE-FM followed a normal distribution, non-parametric-tests were used. For the subscales of UE-FM the same statistical procedure was applied.

**Table 2:** Clinical outcome measures at end of treatment and at follow-up

Within-group analysis							Between-group analysis	
Mean (SD) - Median 95 % confidence interval for the mean (lower and upper bound)							*p*-values	
	End of treatment	Change from	*p*-values	Follow-up	Change from	*p*-values	Change from	Change from
	T1	baseline to T1		T2	baseline to T2		baseline to T1	baseline to T2
UE-FM								
*EG*	38.33 (17.30) – 39	6.00 (6.31) – 4	**.008**	46.22 (14.96) – 52	13.89 (9.88) – 10	**.004**		
	[25.04–51.63]	[1.15–10.85]		[34.72–57.73]	[6.29–21.48]			
							.715	**.037**
*CG*	43.22 (12.62) – 44	6.33 (4.50) – 7	**.008**	41.78 (12.47) – 40	4.89 (4.31) – 5	**.016**		
	[33.52–52.92]	[2.87–9.79]		[32.19–51.36]	[1.57–8.20]			
CAHAI								
*EG*	33.56 (15.08) – 36	1.00 (1.66) – 0	.125	25.11 (16.04) – 42	2.56 (4.64) – 1	.094		
	[21.96–45.15]	[-0.27–2.27]		[22.78 – 47.44]	[-1.01–6.12]			
							.553	.552
*CG*	34.22 (14.71) – 43	0.89 (2.37) – 0	.500	34.89 (14.34) – 43	1.56 (3.64) – 0	.250		
	[22.91–45.53]	[-0.93–2.71]		[23.87–45.91]	[-1.25–4.36]			
BI								
*EG*	85.56 (10.90) – 88	0.22 (0.67) – 0	1.000	87.78 (8.27) – 90	2.44 (5.18) – 0	.125		
	[77.18–93.93]	[-0.29–0.73]		[81.42–94.14]	[-1.53–6.42]			
							1.000	.241
*CG*	91 (6.69) – 90	0.44 (1.33) – 0	1.000	91 (6.69) – 90	0.44 (1.33) – 0	1.000		
	[85.86–96.14]	[-0.58–1.47]		[85.86–96.14]	[-0.58–1.47]			
Hamilton								
*EG*	13.89 (9.61) – 8	-0.56 (1.13) – 0	.500	13.67 (9.85) – 8	-0.78 (1.39) – 0	.250		
	[6.50–21.28]	[-1.42–0.31]		[6.10–21.24]	[-1.85–0.29]			
							.506	.776
*CG*	10.78 (10.15) – 5	-1.67 (2.83) – 0	.250	11.67 (11.87) – 5	-0.78 (3.93) – 0	.688		
	[2.98–18.58]	[-3.84–0.51]		[2.54–20.79]	[-3.80–2.24]			

Bold values indicate significant values (*p* <.05), *p*-values for within-group analysis were obtained with Wilcoxon signed-rank test, *p*-values for between-group analysis were obtained with Wilcoxon rank-sum test

**Fig. 4:**
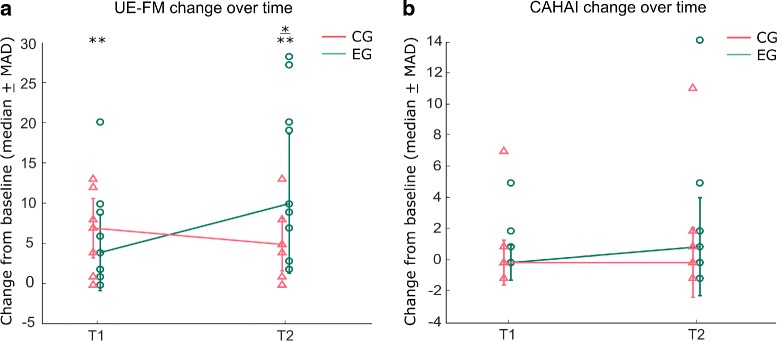
Clinical measurements. Change in UE-FM (**a**) and CAHAI (**b**) from baseline to the end of treatment at week 6 (T1) and to follow-up at week 12 (T2) (i.e. 6 weeks after the end of the treatment) for the experimental (*EG*, *green*) and the control group (*CG*, *red*). Error bars indicate median absolute deviations for each group. The individual data for each subject is indicated with triangles for CG and with circles for EG

**Fig. 5:**
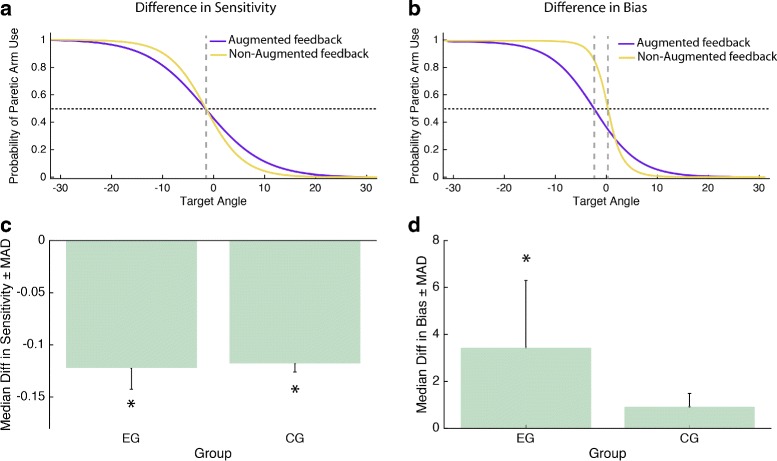
Influence of the augmented sensorimotor feedback on hand selection. **a**-**b** Psychometric functions describing hand selection patterns of two representative patients in the EG group. The *purple line* describes the probability of using the paretic limb in the Whack-a-Mole training scenario. The *yellow line* refers to arm use during the virtual evaluation scenario, when no augmented sensorimotor feedback was provided. Panel **c** indicates a difference in the sensitivity to the target position between scenarios (i.e. different slopes). Panel **d** presents a difference in bias (i.e. change in the Point of Subjective Equality between scenarios)

In order to determine a change in hand selection patterns we first fitted the probabilities of selecting the paretic limb to a psychometric function for discrimination. Calculating the 50 % intersection point of the function provided us with the point of subjective equality (PSE). PSE represents an angle in space at which the patient demonstrates an equal probability to reach with one or the other limb (Fig. 4). We extracted the PSE and the slope of the psychometric function for every patient within the Whack-a-mole, the virtual evaluation and the real world scenario for every session. A change in PSE would reflect a change in hand selection bias, whereas a change in the slope indicates a shift in sensitivity for certain target locations (Fig. 5a-b).

In order to explore whether the patient’s reinforcement history could influence arm use, we performed a sequential analysis of hand bias. We computed the patients probability to select the paretic limb in each trial in respect to either the outcome (success or failure), effector selected (paretic or non-paretic) or a combination of the two factors in the previous trial. We then compared the probabilities of the individual factors or their combinations within and across group. These two categorical values were obtained for each patient in the Whack-a-mole and virtual evaluation scenario for each session. If normality was confirmed by the Lilliefors test, a dependent or independent *t*-test was performed to compare the factors within or across group, otherwise a Wilcoxon signed-rank or Wilcoxon rank-sum test was applied.

Effect sizes (Pearson’s *r*) for each for non-parametric test were calculated as follows:

$$\begin{array}{@{}rcl@{}} &r &= \frac{Z}{\sqrt[]{N}} \end{array} $$

where *Z* is the *z*-score of the non-parametric statistic performed and *N* is the total number of observations. The effect sizes for each parametric test (*t*-tests) were calculated as follows:

$$\begin{array}{@{}rcl@{}} &r &= \sqrt[]{\frac{t^{2}}{t^{2}+df}} \end{array} $$

Statistical analysis was performed with MATLAB R2015b and IBM SPSS Statistics Data Editor (Version 19).

## Results

### Clinical impact

In order to explore the efficacy of RIMT on motor recovery, the clinical outcomes before and after the intervention were compared and analyzed. The within-group analysis indicated a significant change from baseline in our primary outcome UE-FM at T1 and T2 for EC (*p*=.008, *r*=−.595 and *p*=.004, *r*=−.628 respectively) and CG (*p*=.008, *r*=−.596 and *p*=.016, *r*=−.560 respectively) as shown in Table 2 and Fig. 5. The between-group analysis revealed in addition a significant difference in UE-FM change at T2 (*p*=.037, *r*=.479). This suggests that EG achieved significant higher UE-FM scores at T2, whereas the measurement at T1 and baseline was not significantly different between the groups. No further significant within- or between-group changes were found in the other clinical measurements.

The analysis of the subscales of UE-FM revealed significant effects at within-group level. UE-FM-Proximal change was significant at T1 and at T2 for EC (*p*=.016, *r*=−.560 and *p*=.004, *r*=−.629 respectively) and CG (*p*=.016, *r*=−.558 and *p*=.016, *r*=−.558 respectively, as shown in Table 3. Further the improvement for UE-FM-Wrist was significant for EG at T2 (*p*=.016, *r*=−.572). The remaining subscales changes revealed no significant within- or between-group improvements.

**Table 3:** UE-FM subscales outcome measures at end of treatment and at follow-up

Within-group analysis							Between-group analysis	
Mean (SD) - Median 95 % confidence interval for the mean (lower and upper bound)							*p*-values	
	End of treatment	Change from	*p*-values	Follow-up	Change from	*p*-values	Change from	Change from
	T1	baseline to T1		T2	baseline to T2		baseline to T1	baseline to T2
Total UE-FM								
*EG*	38.33 (17.30) – 39	6.00 (6.31) – 4	**.008**	46.22 (14.96) – 52	13.89 (9.88) – 10	**.004**		
	[25.04–51.63]	[1.15–10.85]		[34.72–57.73]	[6.29–21.48]			
							.715	**.037**
*CG*	43.22 (12.62) – 44	6.33 (4.50) – 7	**.008**	41.78 (12.47) – 40	4.89 (4.31) – 5	**.016**		
	[33.52–52.92]	[2.87–9.79]		[32.19–51.36]	[1.57–8.20]			
UE-FM-Proximal								
*EG*	21.00 (8.90) – 18	4.00 (3.57) – 4	**.016**	24.11 (7.67) – 27	7.11 (4.65) – 8	**.004**		
	[10.25–25.75]	[-0.38–7.63]		[21.38–32.63]	[-1.01–6.12]			
							.619	.420
*CG*	24.22 (6.50) – 24	5.33 (4.80) – 4	**.016**	24.33 (7.23) – 24	5.44 (5.30) – 4	**.016**		
	[18.13–29.88]	[-0.75–8.75]		[17.62–30.38]	[4.63–11.38]			
UE-FM-Wrist								
*EG*	7.22 (3.31) – 9	1.44 (2.07) – 1	.063	8.33 (2.00) – 9	2.56 (2.35) – 1	**.016**		
	[6.89–11.13]	[0.0–2.0]		[7.63–10.38]	[-1.13–3.13]			
							.375	.350
*CG*	5.44 (2.92) – 5	0.67 (1.94) – 0	.500	6.22 (2.77) – 5	1.44 (2.40) – 1	.156		
	[3.38–6.63]	[-0.63–0.63]		[2.88–7.13]	[-0.75–2.75]			
UE-FM-Hand								
*EG*	8.44 (5.36) – 9	1.00 (2.29) – 0	.250	9.33 (4.64) – 10	1.89 (4.01) – 1	.250		
	[5.13–12.88]	[-0.50–0.50]		[7.38–12.63]	[-0.25–2.25]			
							.116	.055
*CG*	10.22 (2.63) – 11	-1.22 (3.46) – 0	.500	9.89 (3.79) – 12	-1.56 (3.94) – 0	.313		
	[8.50–13.50]	[-0.63–0.63]		[9.50–14.50]	[-1.37–1.37]			
UE-FM-Coordination								
*EG*	2.89 (1.83) – 4	0.33 (1.00) – 0	1.000	3.00 (1.94) – 4	0.44 (1.01) – 0	.500		
	[2.75–5.25]	[0.00–0.00]		[2.63–5.38]	[-1.25–1.25]			
							.294	.587
*CG*	3.22 (1.48) – 3	0.44 (0.53) – 0	.125	3.44 (1.81) – 3	0.67 (1.00) – 0	.125		
	[2.38–3.63]	[-0.50–0.50]		[1.88–4.13]	[-0.50–0.50]			

Bold values indicate significant values (*p*<.05), *p*-values for within-group analysis were obtained with Wilcoxon signed-rank test, *p*-values for between-group analysis were obtained with Wilcoxon rank-sum test

### Hand selection patterns and effects in arm use

In order to analyze which factors of the training might have contributed to the significant improvement in UE-FM for EG, we extracted and analyzed the factors that influenced hand selection patterns in the intervention scenarios. We observed a strong correlation (*p*<.05, Spearman *r*>.4) between the PSEs measured in the three scenarios (Whack-a-mole, virtual evaluation and real world evaluation) indicating a similar change in arm selection patterns. In addition, sensitivity to target location, as indicated by the slope of the psychometric fit, was significantly lower (median −.12, MAD.041, *p*<.01, Wilcoxon signed-rank test, *r*=−0.88) during the Whack-a-mole scenario for both groups, where feedback augmentation was given to EG, as compared to the virtual evaluation or the real world evaluation scenario where no feedback augmentation was given. Interestingly when the augmented visual feedback was present (i.e. Whack-a-mole scenario), arm use increased significantly, reflected by a positive change in PSE values (median 3.45, MAD 8.53, *p*<.05, Wilcoxon signed-rank test, *r*=.77). CG, who did not experience the feedback augmentation, did not show this effect (median 0.93, MAD 1.67, *p*>.05, Wilcoxon signed-rank test, *r*=.61).

**Fig. 6:**
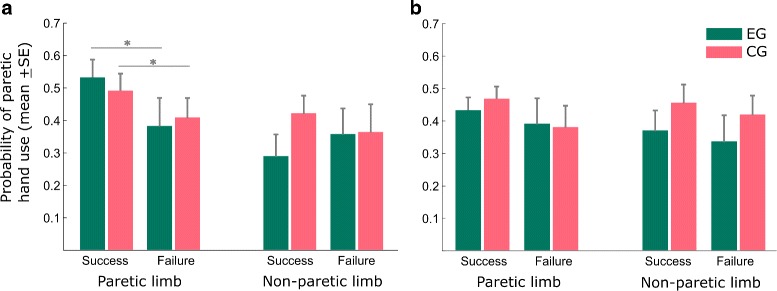
Sequential analysis of hand choice. Influence of hand choice and reinforcement history on arm use. Probability of using the affected arm in the virtual evaluation (no augmentation, **a**) and the Whack-a-mole scenario (augmented sensory feedback for EG, **b**) given the movement outcome (i.e. success or failure) and the hand used (i.e. paretic or non-paretic) in the previous trials (t-1). Error bars indicate standard error of the mean

Hand choice and reinforcement history may influence as well hand selection patterns. We therefore investigated the contribution of these factors to arm use, and assessed the probability to select the paretic hand in trial t, dependent if in the previous trial t-1 A) the paretic or non-paretic limb was selected, B) the outcome was successful or a failure, or C) combinations of these two events occurred. The sequential analysis revealed that in the virtual evaluation scenario the factors outcome or selection alone did not seem to influence decision making in the next trial, but the combination of the two factors led to significant effects. When the patients used their paretic limb and succeeded to reach for the target, the probability to select the paretic limb again in the next trial was higher than in the case of failure. Moreover this effect was more pronounced for CG than for EG (for EG *p*=.044, *r*=.721, paretic/success mean 0.529±0.163 SD, paretic/failure mean 0.380±0.257 SD; for CG *p*=.006, *r*=.795, paretic/success mean 0.489±0.155 SD, paretic/failure mean 0.406±0.178 SD, Fig. 6a). In contrast was this sensitivity for movement outcome not present when EG experienced the augmentation of goal-oriented movement, e.g. in the Whack-a-mole scenario, (for EG *p*=.349, *r*=.332, paretic/success mean 0.431±0.118 SD, paretic/failure mean 0.390±0.234 SD, Fig. 6b), whereas the sensitivity of the control group slightly failed to be significant (for CG *p*=.057, *r*=.618, paretic/success mean 0.466±0.114 SD, paretic/failure mean 0.380±0.195 SD, Fig. 6b). Both groups showed no sensitivity when using the non-paretic arm. As of the reported results did not violate the assumption of normality, *t*-tests were applied.

## Discussion

In this study we examined the effects of providing augmented sensorimotor feedback of goal-oriented arm movements on motor recovery and arm use after stroke. We named this combined treatment “Reinforced Induced-Movement Therapy” (RIMT). Simulations from a model of recovery after stroke support that reinforcement-based therapies can be combined with mild-restriction of the less-affected arm use to maximize recovery. We tested this assumption by conducting a double-blind randomized controlled trial on chronic stroke patients. Although both groups of patients showed motor recovery at the end of the treatment, only patients who underwent RIMT rehabilitation protocols experienced further functional gains during the follow-up period. Interestingly, these gains in the RIMT group were accompanied by an increased arm use during training. These results emphasize the benefits of providing augmented implicit reinforcement on motor recovery and arm use.

Psychosocial factors are often neglected in the study of rehabilitation, however they might be critical ingredients in successful recovery [41–45]. A model of recovery proposed by Folkman and Lazarus et al. hypothesizes that suboptimal outcomes may worsen due to self-limiting cognitive believes, further leading to poor coping strategies and initiating a vicious loop of recovery in which adaptive responses, stress, and function degrade recursively [46]. For instance, adaptive levels of challenge and feedback of progress may result in reduced stress, enhanced self-esteem, and increased self-efficacy [17, 47]. Similarly previous work suggested that learned helplessness affects self-efficacy in a way that the patient over-generalizes the effect that the injury has to the ADLs. As a consequence the patients fails to test and update his self-limiting believes as he or she thinks of not being able to perform day-to-day activities [48]. Our results revealed that hemiparetic stroke patients exhibit a pronounced sensitivity to success and failure when using the affected arm, which strongly biases arm use. Similar findings have been reported in previous experiments [21]. Surprisingly, we also found that when we provided visuomotor feedback of goal-oriented arm movements, this sensitivity disappeared. The combination of explicit and implicit reinforcement in RIMT protocols may be the key factor for changing the patient’s perceived competence, leading to sustained improvements in arm use and rising the intensity of the training. Furthermore, we speculate that frequent and sustained exposure to RIMT goal-oriented movement augmentation may be able to condition the patient to incorporate the affected limb into performance of ADLs. Future experiments will validate this hypothesis and evaluate the impact and the retention of these effects in domiciliary setups.

It has to be noted that previous studies investigating VR-based rehabilitation protocols do not examine whether the observed effects continue to persist during follow-up periods after the intervention ends [49], which could be one of the reasons why the efficacy of VR-based clinical intervention is still debated and meta-analyses that determine a clearly proven effectiveness of these interventions are basically non-existent [50, 51]. Our results showed that, three months after the therapy ended, both groups retained the therapy-induced motor gains. Surprisingly, the EG group exhibited a significant improvement in motor function during the follow-up period. We find this result encouraging as it might indicate that the benefits of RIMT, if driven by behavioral changes, may be sustained in time. It has been previously shown that a 10-point change in UE-FM corresponds to a 1.5-point change in measurements of functional independence (FIM) [52], which constitutes the Minimally Clinically Important Difference. FIM is a standardized assessment of the patients ability in performing the activities of daily living independently. The EG in our study showed a mean improvement in UE-FM at follow-up of almost 14 points, which might possibly correspond to a functional gain in the performance of ADLs.

This study faces several limitations that have to be considered. First, the computational model used does not fully implement all defined training methods of RIMT and CIMT (see Additional file 1). Only the restriction of the less-affected arm and augmented reinforcement was simulated. Other factors such as shaping through increasing difficulty and therapist feedback as well as adherence promotion were not taken into account. An important limitation of this the model is that it only simulates bi-stable recovery patterns (improvement or regression). Cases of patients that show neither improvement nor deterioration were not considered. Second, the sample size used in the clinical evaluation was small and contained a considerably high individual variability, therefore reducing the overall statistical power of our results. In this regard, we also were not able to answer yet the question, whether RIMT would be suitable for patients with severe hemiparesis as the selection criteria was stringent in order to minimize inter-subjects variability. However, since RIMT does not necessarily include distal movements in its training protocols, it is also suitable for those patients who do not present sufficient range of movement in the metacarpophalangeal and interphalangeal joints to benefit from CIMT [5, 10]. Moreover, the total exposure to training in RIMT is remarkably inferior to the exposure time delivered by Reduced-intensity mCIMT [10], to the best of our knowledge the most reduced form of CIMT found in literature. In respect thereof future clinical trials with larger sample sizes are required to validate our results and determine which type of patients could benefit the most from RIMT protocols. We further propose that prospective studies should consider to test our findings directly against fully incorporated CIMT trials, as we investigated specifically the effect of augmented visuomotor feedback of goal-oriented arm movements against a control group without augmentation, prioritizing to minimize confounding variables and to guaranty a double-blinded experimental design.

## Conclusions

In this study we propose and validate a novel technique for motor recovery: “Reinforced Induced-Movement Therapy” (RIMT). This therapy exposes the patient to augmented goal-oriented arm movements in VR, and combines customized intensity training with implicit and explicit reinforcement to boost arm use and motor improvement. Our results show that after six weeks of daily training with RIMT, patients continue to experience further gains until week 12 follow-up, a period in which patients did not receive any specific training. The control group did not show such effects. We also found a significant increase in the paretic arm use during RIMT sessions.

These findings are in line with simulations from a computational model, which support that mild restriction of the less-affected limb paired with RIMT reinforcement strategies to promote the usage of the paretic limb could lead to an effective rehabilitation approach. By incorporating psychosocial attributes into the rehabilitation approach, RIMT may be a powerful mechanism to shape the patient’s perceived competence, reinforce non-compensatory behavior, and overcome learned non-use.

## Abbreviations

ADL, activities of daily living; BI, barthel index; CAHAI, chedoke arm and hand activity inventory; CIMT, constraint-induced motor therapy; CG, control group; DAC, distributed adaptive control; EC, experimental group; FIM, functional independence measure; MNS, mirror neuron system; PSE, point of subjective equality; RGS, rehabilitation gaming system; RIMT, reinforcement-induced motor therapy; UE-FM, upper extremity fugl-meyer assessment; VR, virtual reality

## Additional file

Additional file 1 Methodology of computational model. Detailed explanation of the implementation of the computational model used in this study. (PDF 1064 kb)

Additional file 2 CONSORT flow diagram. Flow diagram of the process of randomisation of trial. (PDF 206 kb)
