# Supplementary material for: Counteracting learned non-use in chronic stroke patients with reinforcement-induced movement therapy
Source: J Neuroeng Rehabil. 2016 Aug 9;13:74. doi: 10.1186/s12984-016-0178-x (PMC4979116; doi:10.1186/s12984-016-0178-x)
Supplement: Additional file 1 — Methodology of computational model. Detailed explanation of the implementation of the computational model used in this study. (PDF 1064 kb) [file 12984_2016_178_MOESM1_ESM.pdf]

RESEARCH

Supplementary Materials: Modeling and simulation of motor recovery in stroke patients

Belén Rubio Ballester[1†], Martina Maier[1†], Rosa María San Segundo Mozo[2], Victoria Castañeda[2], Armin Duff[1] and Paul F.M.J. Verschure[1,3*]

*Correspondence: paul.verschure@upf.edu
[1]Laboratory of Synthetic, Perceptive, Emotive and Cognitive Systems, Universitat Pompeu Fabra, Roc Boronat 138, Barcelona, Spain
Full list of author information is available at the end of the article
†Equal contributor

This file includes:
- Supplementary Text
- Figures S1 to S4
- References

In the main text, we introduced a computational model of motor recovery after stroke that describes the close interaction between arm use and motor improvement. In the next sections we provide a detailed description of the implementation of this model and we present preliminary results from simulations.

Computational Model description

In reaching movements, movement extent and direction have different sources of variable and systematic errors [1, 2], suggesting that hand paths are initially planned in vectorial coordinates without taking into account joint motions. Our model comprises a motor unit formed by two lateralized motor networks of direction-tuned cells, which generate vectorial planed movement trajectories, with specific extent and direction (Figure S1). The activity of each motor neuron is determined by the difference between the preferred direction of neuron i θ_p^i and the desired movement direction θ_d [3, 4]:

$$y = [cos(\theta_d - \theta_p^i) + N(0, {\sigma_{SDN}}^i)]^+ \tag{1}$$

$$where[x]^+ = \begin{matrix} x, if x > 0 \\ 0, if x < 0 \end{matrix}$$

The angle for the planed trajectory is the vector sum of the activity of each neuron i, and follows the same method as reported in [3]. In the network that codes movement extent, the length of the planed movement trajectory is determined by the weighted sum of activity of each neuron i in the network. Each weight w_i updates after execution as a function of error in extent given the activity of neuron i, rapidly adjusting to the optimal extent of an angle dependent trajectory:

$$\delta w_i = \eta(X_e - X_d)y_i(\theta_d, \theta_{p,i}). \quad (2)$$

where η is a learning rate, X_e is the executed movement extent, and X_d is the desired movement extent (i.e. the actual extent needed to reach the target position). Each of these networks contained 20 neurons, and the learning rate η was set at 0.5.

After execution, a combination of error-based and use-dependent learning mechanisms [3] updates the motor cortex contra-lateral to the selected limb, modulating the preferred angles of motor neurons to enhance performance accuracy in the future execution of similar movements. Each of this networks contained 500 neurons with uniformly distributed direction sensitivities (0-360 degrees). For the parameters in this network we used the same values reported in [3].

Estimation of Energies

Planed trajectories in each hemisphere are transformed to intrinsic coordinates for estimating their biomechanical cost (i.e. energy). In the last decade, several authors referred to the general hypothesis that the nervous system optimizes performance as a function of energy expenditure [5, 6, 7]. Following this line, a previous study showed that humans prefer to select reaching movements which are biomechanically easier to perform [8]. This implies that the brain codes information about the future biomechanical costs of multiple movements, before deciding which one to execute. To account for this bias in action selection an independent unit in our model estimates the energies needed to achieve each planned joint angle. First, we add a unit in the motor cortex which performs the transformation of planed movements from vectorial coordinates to intrinsic coordinates. In order to compute the interaction torques produced at each joint by motions of upper arm and forearm segments we use the method described in [9] modified to account only for planar reaching movements with fixed shoulder positions (i.e. constrained trunk movements). Interaction torques for shoulder (T_s) and elbow (T_s) for each limb h (i.e. left or right) are given by:

$$T_{h,s} = \alpha\ddot{\theta}_s + \beta\ddot{\theta}_e + \gamma\dot{\theta}_e^{\,2} - 2 * \gamma\dot{\theta}_s\dot{\theta}_s \quad (3)$$

$$T_{h,e} = \epsilon\ddot{\theta}_e + \beta\ddot{\theta}_s + \gamma\dot{\theta}_s^{\,2}$$

where

$$\alpha = m_s r_s^2 + I_s + m_e[l_s^2 + r_e^2 + 2l_s r_e \cos(\theta_e)] + I_e$$

$$\beta = m_e l_s r_e \cos(\theta_e) + m_e r_e^2 + I_e$$

$$\gamma = m_e l_e r_e \sin(\theta_e)$$

$$\epsilon = m_e r_e^2 + I_e$$

This transformation needs to take into account the anthropometric properties of the moving arm (mass of upper-arm m_s, mass of forearm m_e, center of mass of upper arm r_s and forearm r_e, length of upper arm l_s and forearm l_e, and moments of inertia at center of mass of the upper arm I_s and forearm I_e). We used anthropometrical data from [10].

The expected energies for a specific action a are computed as the sum of the multiplication of angular velocity ($\dot{\theta}$) and torques (T) for shoulder and elbow at each time step t until the final time step n:

$$E_a = \sum_{t=0}^{n} T_s(t)\dot{\theta}_s(t) + T_e(t)\dot{\theta}_e(t) \quad (4)$$

Action Selection

The action selection module is a variation of an interactive race model [11] divided in two competing units corresponding to two possible actions (i.e. reaching with the left limb or with the right limb). Each of them recursively accumulates action-specific activity A_h for each hand h given the expected reward of this action. In addition, it is recursively inhibited by the competing action network activity. As a result, targets appearing at left or right workspace will show higher probability of being reached by the ipsilateral hand. In addition, a noise term, N, is added to represent noise in input to account for exploration in action selection. Activity ($A_{a,h}$) for action a (i.e. joint rotations for reaching towards an specific location) and limb h is given by:

$$A_{a,h}(t) = A_{a,h}(t-1) + s_r Q_{a,h} - s_e E_{a,h} - s_a A_{a,h^{-1}}(t) + N(t) \quad (5)$$

where $Q_{a,h}$ is the expected reward, and $A_{a,h^{-1}}$ is the accumulated activity for the competing action (i.e. selecting the other hand). s_r, s_e, and s_a are scalars for the expected reward, expected energies and activity of the competing action respectively. This accumulation of activity in the action selection module simulates an increase in striatal dopamine, which outputs an action choice after reaching a threshold [12].

After action execution, the expected reward for the selected movement is updated in memory given the reward prediction error, that is, the difference between the actual reward R and the expected reward Q retrieved from memory [3]. These updates minimize the square of the reward prediction error. For the acquisition of expected rewards Q we used the same methods and parameters reported in [3]. Scalars used in the action selection rule were s_r= 0.4, s_e= 100, and s_a= 0.7. Scalar values were found to provide greater influence for action selection to the expected reward, followed by expected energies and the activity of the competing action. Noise in the interactive race model is normally distributed, with zero mean and standard deviation 0.15, and threshold for action selection was set to 1.

Results from the model

We implemented a computational model of hand selection to study the effect of different CIMT and RIMT therapy combinations on arm use and functional recovery.

First, we ran simulations of 2000 trials of training in order to obtain a model of a healthy subject. As in [3], we simulated a stroke by removing those direction-tuned cells in the motor unit that were sensitive to a specific set of angles (0-90 degrees) corresponding to the upper right workspace. Next, we provided 500 additional trials of training (Figure S4). We observed that immediately after neural removal, the accuracy of executed movements towards targets appearing at the upper-right workspace was affected, reaching a mean directional error of 30 degrees. At this stage, we simulated various combinations of two different treatments, CIMT and RIMT. The first combination variable consisted of forcing the use of the paretic limb with a certain probability (from 0 to 0.9), and the second variable consisted of amplifying the visuomotor feedback with a certain factor (from -0.9 to 0.9). The negative or positive sign of this factor indicates a reduced or an increased directional error feedback respectively. In all simulations, the amplification factor G was fixed to 0.5 (see Equation 1), which reduced the extent of the movement (i.e. length of the movement trajectory) required to reach the target location to half. The model adjusted accordingly to simulate shorter trajectories, consequently reducing the associated expected energy.

We used this model of recovery to simulate the effects of 190 combinations of rehabilitation therapies (10 variations of CIMT x 19 variations of RIMT) on hand selection patterns and performance in accuracy. Each therapy consisted of 3000 trials of training plus 2500 follow-up trials with no treatment. Results show that CIMT alone (0.9 CIMT and 0 RIMT) would promote paretic arm use (Figure S2). However, when both therapies are combined (0.3 CIMT and 0.7 RIMT) equal results can be achieved. We observed very similar effects in the relearning of motor control (Figure S3), showing that those treatments that were most effective in promoting arm use did also induce the greater functional recovery. Contrarily, the amplification of error feedback discouraged the selection of the paretic limb and hampered recovery. In addition, preventing error-based learning by reducing error feedback in 90% impeded recovery.

We performed an analysis of arm use and motor function in a trial-by trial basis to further explore the model's dynamics under four representative conditions: no therapy, pure CIMT, pure RIMT, and a combination of 30% CIMT and 70% RIMT (CIMT+RIMT). Results show that the averaged probability of choosing the paretic limb and mean directional errors across trials (within a 500 trial-window) Figure S3 increased in all treatment conditions. We identified a threshold of performance (15 degrees of directional error), that initiated a virtuous loop of recovery by promoting spontaneous use of the paretic limb. This bistable dynamics induced further performance improvement and restored typical hand selection patterns at follow-up. Contrarily, the no-therapy condition progressively discouraged the use of the affected limb and predicted further deterioration.

Finally, we used this modeling approach to explore the potential of these representative rehabilitation conditions for inducing cortical reorganization (Figure S4). Early simulated training trials mimicked a healthy subject with uniformly distributed direction-tuned cells. In the left cortex, these distribution showed a bias for recruiting more neurons sensitive to directions corresponding with the workspace ipsilateral to the contralateral limb (i.e. right limb). After inducing the stroke,

all treatment conditions induced positive cortical changes by redistributing intact neurons for covering those movement directions affected by the lesion. After the follow-up period, the distribution of direction-tuned cells across all possible input angles became more uniform, resembling a healthy subject. Notice however that the total number of cells was reduced. Contrarily, no treatment lead to an atypical distribution of neural resources.

Author details
[1]Laboratory of Synthetic, Perceptive, Emotive and Cognitive Systems, Universitat Pompeu Fabra, Roc Boronat 138, Barcelona, Spain. [2]Hospital Universitari Joan XXIII de Tarragona, c/ Dr. Mallafrè Guasch 4, Tarragona, Spain. [3]Institució Catalana de Recerca I Estudis Avançats (ICREA), Barcelona, Spain.

References
1. Ghilardi, M.F., Gordon, J., Ghez, C.: Learning a visuomotor transformation in a local area of work space produces directional biases in other areas. Journal of neurophysiology **73**(6), 2535–2539 (1995)
2. Vindras, P., Desmurget, M., Prablanc, C., Viviani, P.: Pointing errors reflect biases in the perception of the initial hand position. Journal of neurophysiology **79**(6), 3290–4 (1998)
3. Han, C.E., Arbib, M.A., Schweighofer, N.: Stroke rehabilitation reaches a threshold. PLoS computational biology **4**(8), 1000133 (2008). doi:10.1371/journal.pcbi.1000133
4. Todorov, E.: Cosine tuning minimizes motor errors. Neural computation **14**(6), 1233–1260 (2002). doi:10.1162/089976602753712918
5. Todorov, E.: Optimality principles in sensorimotor control. Nature Neuroscience **7**(9), 907–915 (2004). doi:10.1038/nn1309
6. Körding, K.P., Ko, K.P., Wolpert, D.M.: Bayesian decision theory in sensorimotor control. Trends in cognitive sciences **10**(7), 319–26 (2006). doi:10.1016/j.tics.2006.05.003
7. Cisek, P.: Making decisions through a distributed consensus. Current Opinion in Neurobiology **22**(6), 927–936 (2012). doi:10.1016/j.conb.2012.05.007
8. Cos, I., Bélanger, N., Cisek, P.: The influence of predicted arm biomechanics on decision making. Journal of neurophysiology **105**(March 2011), 3022–3033 (2011). doi:10.1152/jn.00975.2010
9. Bagesteiro, L.B., Sainburg, R.L.: Handedness : Dominant Arm Advantages in Control of Limb Dynamics. Journal of neurophysiology **88**(5), 2408–2421 (2009). doi:10.1152/jn.00901.2001
10. Tee, K.P., Burdet, E., Chew, C.M., Milner, T.E.: A model of force and impedance in human arm movements. Biological Cybernetics **90**(5), 368–375 (2004). doi:10.1007/s00422-004-0484-4
11. Wong, K.-F.: A Recurrent Network Mechanism of Time Integration in Perceptual Decisions. Journal of Neuroscience **26**(4), 1314–1328 (2006). doi:10.1523/JNEUROSCI.3733-05.2006
12. Wang, X.-J.: Decision Making in Recurrent Neuronal Circuits. Neuron **60**(2), 215–234 (2008). doi:10.1016/j.neuron.2008.09.034. NIHMS150003

Figures

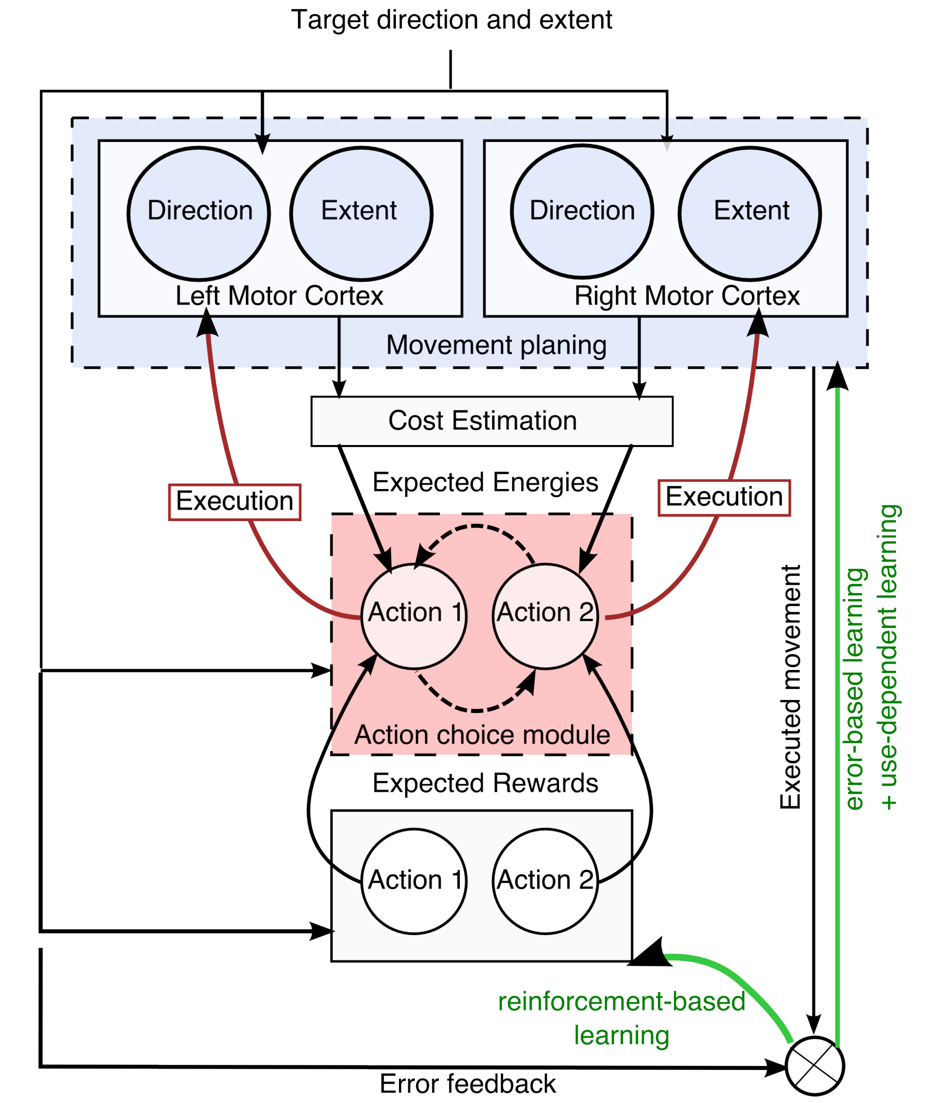

Figure 1 Model diagram. Motor cortex receives the target angle (given by target location at the current trial) as input for movement planing (blue box). Planed trajectories in each hemisphere are transformed to intrinsic coordinates for estimating their biomechanical cost (i.e. energy). Expected energies and expected reward for each planed movement compete for action selection (red box). After execution of the selected action, all networks in the hemisphere contralateral to the selected limb reorganize through error-based learning and use-dependent learning. Expected rewards for the executed action are updated based on actual reward. Green arrows in diagram stand for these three learning mechanisms. Continuous arrows indicate excitatory connections while dashed arrows indicate inhibitory connections.

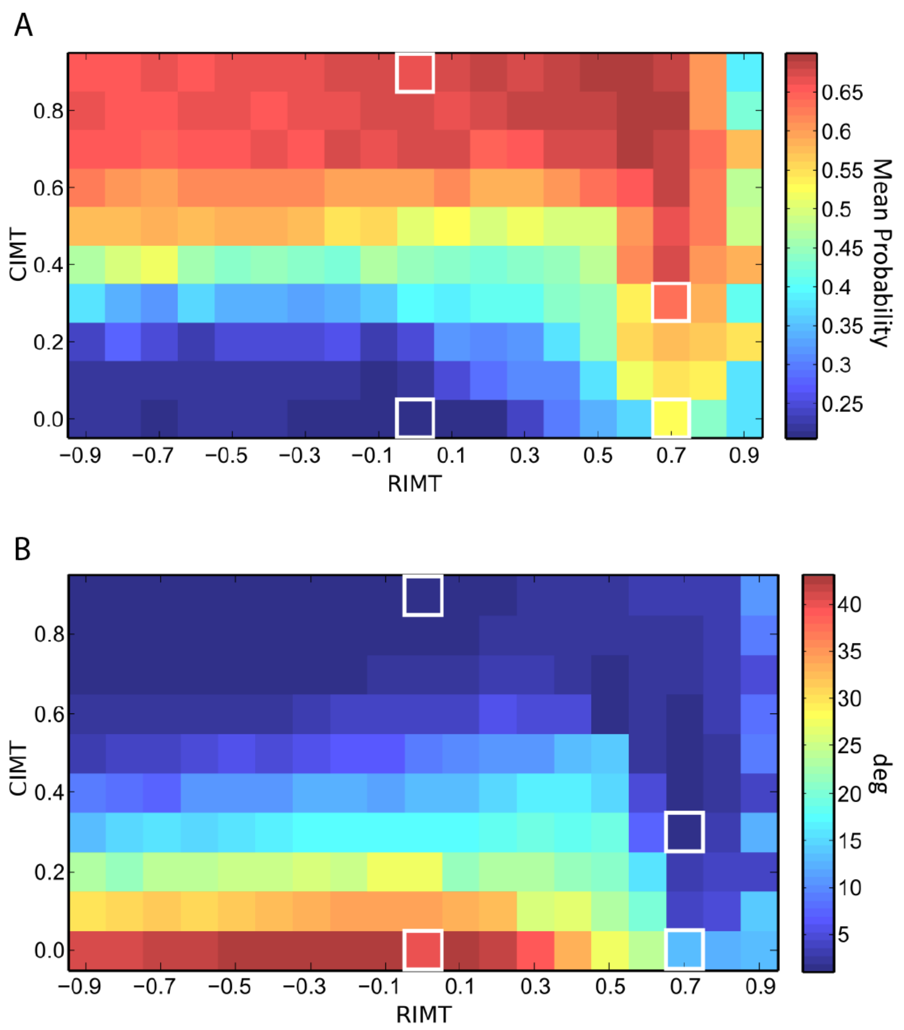

Figure 2 Probability of using the affected arm and directional error simulations. A: Results from simulations showing the probability of selecting the paretic limb (averaged within 500 trials-window), for each treatment condition after follow-up (5500 trials after stroke). B: Results from simulations showing mean directional error of reaching movements executed using the paretic limb for those targets located at the affected workspace. Both results were computed for each treatment condition after follow-up (5500 trials after stroke). X-axis indicates the ratio of directional error suppressed after execution (RIMT conditions). Y-axis indicates the ratio of treatment trials in which the use of the paretic limb was forced (CIMT conditions).

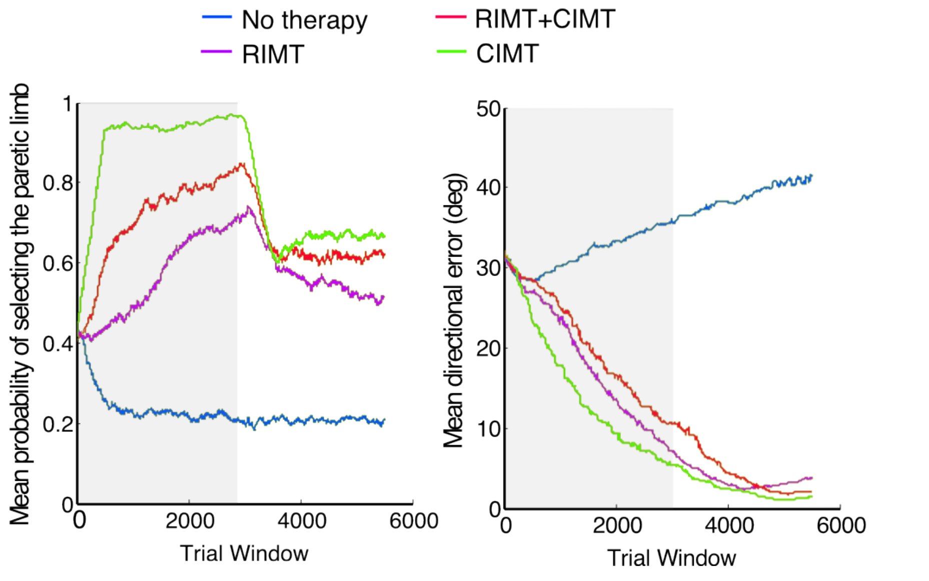

Figure 3 Simulation of therapy outcome. Probability of selecting the paretic limb (left) and averaged directional error for targets appearing in the affected workspace (500 trials-window) for each of the four selected therapies: no specific therapy, CIMT, RIMT, and a combination of CIMT and RIMT protocols. Grey shadow indicates trial windows within the treatment period (3000 trials).

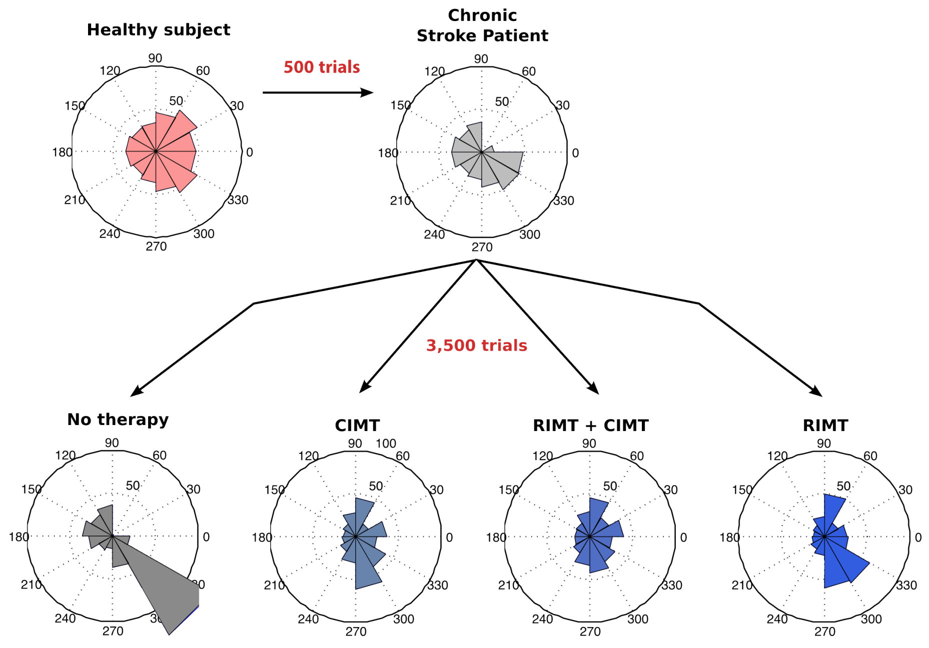

Figure 4 Simulations of Cortical reorganization Cortical reorganization of the left motor cortex for four simulations: after training (healthy subject), after stroke (neural removal), and after follow-up. Each simulation corresponds with one of the four selected treatment conditions: 0.7 RIMT, 0.9 CIMT, the combination of 0.3 CIMT and 0.7 RIMT, and no specific treatment. Polar plots show the distribution of neurons in motor cortex given their preferred direction in angle bins of 30 degrees.
