# Supplementary figures and images for: Counteracting learned non-use in chronic stroke patients with reinforcement-induced movement therapy
Source: J Neuroeng Rehabil. 2016 Aug 9;13:74. doi: 10.1186/s12984-016-0178-x (PMC4979116; doi:10.1186/s12984-016-0178-x)

## CONSORT 2010 Flow Diagram

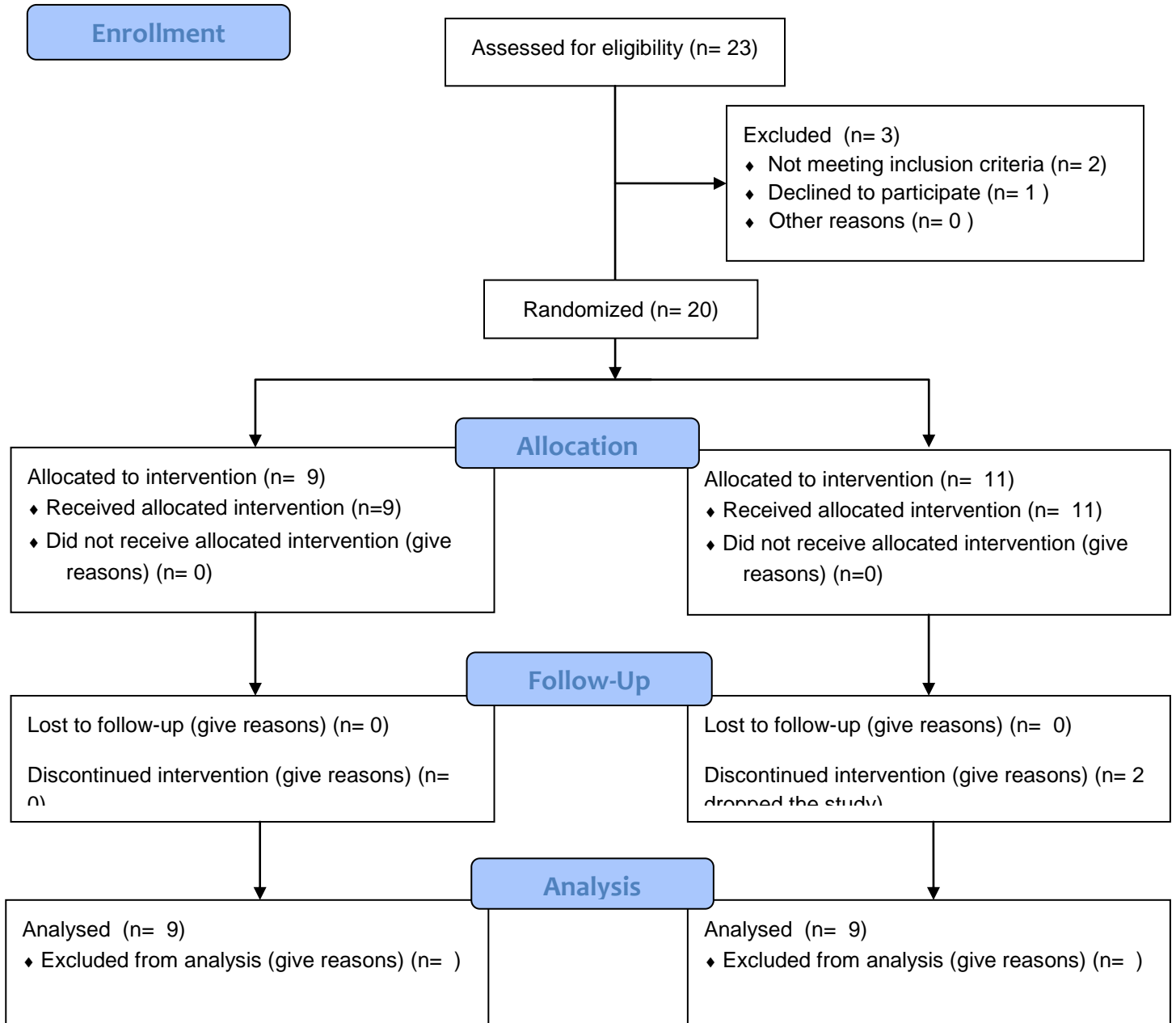

Supplement: Additional file 2 — CONSORT flow diagram. Flow diagram of the process of randomisation of trial. (PDF 206 kb) [file 12984_2016_178_MOESM2_ESM.pdf]
